# Toxic Streptococcal Infection in Children: Report on Two Cases with Uncharacteristic Course of Scarlet Fever

**DOI:** 10.3390/children10030540

**Published:** 2023-03-11

**Authors:** Krystyna Stencel-Gabriel, Dawid Konwant, Karolina Szejnoga-Tułacz

**Affiliations:** 1 Department of Pediatrics, Faculty of Health Sciences in Katowice, Medical University of Silesia in Katowice, 40-055 Katowice, Poland; 2 Doctoral School, Medical University of Silesia in Katowice, 40-055 Katowice, Poland

**Keywords:** streptococcal infection, children, scarlet fever, hospitalization

## Abstract

Introduction: Scarlet fever is usually a mild childhood disease caused by type A *streptococci*. This disease is spread by droplets, mainly through direct contact with an infected person or the objects they have used. In pediatrics, these are significant risk factors for the transmission of infectious diseases. However, it is important to remember the possibility of serious complications in the course of scarlet fever. Aim: This paper provides a discussion of two pediatric cases in order to determine the possibilities of diagnosis, differentiation, and treatment of patients with severe, non-obvious courses of scarlet fever. Methods: The case reports of two patients hospitalized in a pediatric department due to *Streptococcus pyogenes* infection were examined. Results: The patients were admitted to the emergency room with symptoms not directly indicative of type A streptococcal infection, which required further diagnosis. Both patients complained of weakness at the time of presentation. They had an elevated temperature, were dehydrated during the course of gastroenteritis, and passed liquid stools without pathological admixtures. Further stages of diagnosis and treatment required hospitalization in the pediatric department. Therapeutic benefit from the implemented treatment was obtained, and the patients were discharged in good general condition with further recommendations. Conclusions: Medical history, which is often very detailed, can be the key to making the final diagnosis and can supplement the data collected on the basis of laboratory tests. Scarlet fever does not always occur with a mild course, and sometimes its course can be quite non-specific and may require a thorough diagnosis.

## 1. Introduction

*Streptococcus pyogenes* is a bacterium belonging to group A *streptococci* (GAS), which is a significant etiological factor of infections in children, but also among adults. This pathogen causes a number of infections in the pediatric population, including pharyngitis, tonsillitis, scarlet fever, infectious impetigo, pneumonia or subcutaneous tissue inflammation, erysipelas, and toxic shock syndrome. Pharyngitis is one of the milder forms of infection, with approximately 616 million cases diagnosed worldwide each year. Existing statistical data indicate that there are about 163 thousand deaths annually in the world due to invasive streptococcal infection. Thus, it is an important epidemiological risk factor for death and severe complications [1]. Scarlet fever is a common infectious disease with a characteristic morphology; however, according to existing data, its diagnosis is often delayed or not considered at all in groups of children over five years of age due to its similarity to streptococcal pharyngitis [2,3]. Distinguishing between viral and bacterial etiologies of acute pharyngitis can be problematic when attempting to make a correct diagnosis. Current statistics indicate that for acute pharyngitis and acute tonsillitis in children, less than 30% are of bacterial origin [4,5]. The highest peak incidence of GAS pharyngitis is observed during winter months. In addition, an increased number of cases may also occur in spring and summer [4,6].

This paper presents two cases of patients hospitalized in the Department of Pediatrics, Medical University of Silesia, in which the medical interview turned out to be the key to making the final diagnosis of severe, atypical scarlet fever.

## 2. Case Reports

### 2.1. Patient 1

#### 2.1.1. Patient Information

A 4-year-old patient came to the emergency room in the Department of Pediatrics due to dehydration during the course of gastroenteritis and worsening skin lesions. Over the previous three days before admission, the boy had a high and intense fever reaching 41 °C with a mediocre response to antipyretics. He passed numerous liquid stools without pathological admixtures. On the day before hospital admission, a small, maculopapular, and itchy rash was present on the patient’s skin, as well as redness. His lips were dry and cracked. The boy drank water quite willingly, but his diuresis was significantly limited according to his parents’ opinion. The examination showed that the patient and his parents had an upper respiratory tract infection two months before hospitalization, when he received Cefuroxime orally in a dose appropriate for his age. Subsequently, three weeks before hospitalization in the ward, the patient received Clarithromycin in the oral form for 14 days as an outpatient due to pharyngitis. The information obtained from the parents did not indicate that the patient had contracted COVID-19.

#### 2.1.2. Clinical Findings and Diagnostic Assessment

In the emergency room, a physical examination revealed severe signs of dehydration, an average clinical condition, significant weakness (the appearance of a very sick child), and discrete swelling of the hands and feet. Moreover, the patient had chapped redness of the lips, with swelling of the gums and tongue, and cervical lymphadenopathy with predominance on the left side. The patient’s skin showed erythema on the face, and fine-petal rash in the area of the chest, abdomen, back, and buttocks.

The preliminary laboratory tests showed high inflammatory parameters, leukocytosis with neutrophilia, hyponatremia, compensated metabolic acidosis, high urea concentration, and decreased GFR (Table 1). Initially, due to the child’s severe clinical condition and significantly elevated inflammatory parameters, which could indicate severe sepsis, cultures were taken from urine (negative result), feces (negative result), blood (negative result), and from a throat swab. The quantitative test for antibodies on the second day of his stay turned out to be positive, with a result of 141.2 U/mL. During hospitalization, it was hypothesized that the patient might have contracted COVID-19 during the infection two months prior to admission to the ward. Control laboratory tests, including inflammatory parameters, were performed after 72 and 96 h of hospitalization and at the end of therapy, i.e., on the nineth day of therapy (Table 2). On the fifth day, the result of the microbiological examination of the throat was obtained, which showed the presence of *streptococcus pyogenes* in the high titer (++). This bacterial species is sensitive to beta-lactam antibiotics. Thus, penicillin was the drug of choice. During hospitalization, abdominal ultrasound was performed four times, lung ultrasound was performed three times, and lymph node ultrasound was performed once. The first ultrasound examination of the abdominal cavity performed on the second day of hospitalization showed a slight amount of interloop fluid. In the next two follow-up examinations which were carried out on consecutive days, there was no deterioration. The last follow-up examination performed on the 9th day of hospitalization showed a regression of the previously described changes. The first ultrasound of the lungs was performed on the second day of treatment, showing slight bilateral inflammatory consolidations at the bottom of the lungs, which was confirmed in the next ultrasound imaging performed 24 h later, resulting in the diagnosis of bilateral pneumonia in the child. The examination performed on the nineth day of treatment confirmed the regression of the previously described inflammatory changes in the lungs. The cervical and submandibular lymph node examination on the fourth day showed inflammatory lymphadenopathy.

The patient was consulted twice by a cardiologist on the third and tenth days of treatment due to his severe general condition during the initial days of hospitalization. The performed electrocardiographic and echocardiographic examinations showed no significant abnormalities. Despite this, the consulting cardiologist additionally recommended using NT-proBNP, cardiac markers, and daily monitoring of electrocardiogram for the purpose of differential diagnosis of pediatric inflammatory multisystem syndrome temporally associated with SARS-CoV-2 (PIMS-TS). Laryngological consultation was carried out on the third day of hospitalization, during which a strongly reddened throat and swollen purulent deposits on the tonsils were found.

#### 2.1.3. Therapeutic Interventions and Outcome

On the first day of hospitalization, after the laboratory tests, Ceftriaxone at 50 mg/kg/day in the intravenous form was introduced to the treatment, and after the next three days, 10 mg/kg/dose of Vancomycin was also added and administered intravenously four times a day. This drug was implemented due to the lack of improvement after the treatment used so far. It was suspected, due to the lack of a definitive throat swab, that a penicillin-resistant pathogen might be the source of the infection. After the use of third-generation cephalosporin, laboratory tests showed a decrease in inflammatory parameters (Table 2), but the boy’s clinical condition did not improve. In addition, diuresis was limited (400 mL per 24 h). There was swelling of the feet, hands, and the lumbosacral area. The erythematous rash persisted. Apart from pharyngitis and tonsillitis, extensive stomatitis developed in the oral cavity, which prevented the patient from taking liquids and food.

Based on a positive throat swab obtained on day 5 of hospitalization, the patient was diagnosed with scarlet fever. *Streptococcus pyogenes* was grown to a large extent, as well as *Haemophilus parainfluenzae*. On the same day, oral Phenoxymethylpenicillin at 1,000,000 I.U. was added to the treatment twice a day for a period of 10 days, which lasted until the end of hospitalization. Previously administered antibiotics were discontinued. In addition, during hospitalization, the patient was treated intravenously with parenteral hydration (variable values), multi-electrolyte fluid (QD), and Furosemide (BID). The following drugs were administered orally: Spironolactone (QD), Paracetamol (QID), Ibuprofen (to be taken when it was necessary), Clemastine (QD), and a drug containing Magnesia hydroaspartas and Kalii hydroaspartas (to be taken when it was necessary).

#### 2.1.4. Post-Hospital Recommendations

The recommendations included the implementation of home isolation until the full resolution of all clinical symptoms and the performance of control laboratory tests (urinalysis, complete blood count, total protein, CRP, creatine kinase, and transaminase concentration) with outpatient control of the results by the pediatrician. In addition, it was recommended that the patient avoided excessive physical effort and performed daily weight control on an empty stomach, with the implementation of a diet of easily digestible food. A referral was issued for outpatient follow-up visits to nephrology and cardiology outpatient clinics. In the event of recurrent pre-existing clinical symptoms, the child’s mother was advised to report again to the pediatric ward. The total time of the patient’s stay in the hospital as a result of hospitalization was 14 days.

### 2.2. Patient 2

#### 2.2.1. Patient Information

A 4-year-old boy was admitted to the emergency room of the Department of Pediatrics late in the evening due to dehydration during the course of gastroenteritis one day after patient no. 1. Over the previous three days before admission, the boy had a high and intense fever reaching 40 °C with a mediocre response to antipyretics. He was significantly weakened, vomiting, and passing liquid stools without pathological admixtures. In addition, according to the parent’s assessment, diuresis was significantly reduced compared to the previous days. Over the previous two days before hospital admission, a rash was present on the child’s skin, which was diagnosed in an outpatient setting as infectious erythema. During the medical interview, the parents denied having previously contracted COVID-19.

#### 2.2.2. Clinical Findings and Diagnostic Assessment

During the time spent in the emergency room, the physical examination showed abnormalities in addition to signs of increased dehydration; significant weakness (the appearance of a very sick child); and fine-spotted rash on the skin of the face (with a Filatov’s triangle), chest, abdomen, and genital area; edema on the hands and feet; a raspberry red tongue; and a reddened throat with swollen red tonsils. On the second day of hospitalization, the skin rash became “brushy”. Pastia’s lines appeared in the groin and elbow folds, and the Filatov triangle became very pronounced.

Blood, urine, and feces samples were taken. The preliminary laboratory tests showed elevated parameters of inflammation and hyponatremia, and significantly elevated activity of transaminases (Table 1). The quantitative test for antibodies on the second day gave a negative result. Due to the characteristic symptoms, a pharyngeal culture for GAS was not ordered. Control laboratory tests, including inflammatory parameters, were performed after 48 and 96 h of hospitalization and at the end of therapy, i.e., on the 10th day of therapy (Table 2).

During hospitalization, three abdominal and retroperitoneal ultrasounds and two lung ultrasounds were performed. The first abdominal examination was performed on the third day of hospitalization, showing a slight amount of interloop fluid in the right iliac fossa and hepatomegaly, which did not change in the control examination performed after 24 h. Another control examination of the abdomen performed on the eighth day of hospitalization showed a significant improvement in the form of a reduction in the amount of interloop fluid. The first ultrasound examination of the lungs performed on the third day of hospitalization showed the presence of fluid in both pleural cavities, hepatosis of the lower lobe of the left lung, and numerous small consolidations within the right lung. The control lung examination carried out on the eighth day of hospitalization showed a regression of inflammatory changes in the lungs.

On the second and ninth days of hospitalization, the patient underwent cardiological consultations. The performed electrocardiographic and echocardiographic examinations showed no significant deviations from the normal state. Additionally, on the second day of hospitalization, an ENT consultation was carried out, which revealed a vivid red throat, reddened tonsils, and swollen deposits on the back of the throat along with purulent discharge. The performed posteroanterior view (PA) + lateral chest X-ray on the ninth day showed lung areas without focal changes and a silhouette of the heart within normal limits.

#### 2.2.3. Therapeutic Interventions and Outcome

Initially, intensive parenteral hydration was used therapeutically. On the day of admission, the patient was treated with Ceftriaxone at a dose of 50/mg/kg/day in intravenous form, which lasted until the eighth day. On that day, the boy had an intense fever reaching 39 °C and was very weak. He had diuresis, but despite the use of parenteral hydration, it was significantly limited. It amounted to 350 mL per 24 h. Persistent peripheral edema and a rapid enlargement of the abdominal circumference were also noticed.

Due to the characteristic features of scarlet fever on the second day of hospitalization, a diagnosis was made in this regard. On that day, Phenoxymethylpenicillin at 1,000,000 I.U. was administered orally twice a day until the end of hospitalization in a dose appropriate to the patient’s age, along with a probiotic that had a strain of *Saccharomyces boulardii*. The probiotic was used due to continued diarrhea. The following treatments were administered to the patient intravenously: parenteral hydration (variable values), multi-electrolyte fluid (QD), Furosemide (BID), and Paracetamol (BID). The following drugs were used orally: Spironolactone (BID), Ibuprofen (to be taken when it was necessary), Cetirizine hydrochloride (BID), and inhalations with Budesonide (QD).

#### 2.2.4. Post-Hospital Recommendations

The recommendations included home isolation until complete recovery. Control laboratory tests were also recommended about a week later (urinalysis, aminotransferase concentration, complete blood count, total protein, and CRP), together with a consultation at the pediatric clinic. Avoidance of excessive physical effort, daily weight control on an empty stomach, and a diet of easily digestible food were also recommended. A referral for an outpatient follow-up visit to a nephrology clinic was issued. If pre-existing clinical symptoms recurred, the child’s mother was advised to return to the pediatric ward. The total time of the patient’s stay in the hospital was 10 days.

### 2.3. Comparison of Cases

Due to the initially severe clinical condition presented in both boys, the possibility of a systemic inflammatory reaction after recovering from COVID-19 was taken into account. Laboratory diagnostics were extended in accordance with the PIMS-TS protocol with a repetition frequency depending on clinical needs. After the introduction of antibiotic therapy, a significant decrease in inflammatory markers and aminotransferase activity was found in both patients. The concentrations of triglycerides were surprisingly high, while cholesterol and total protein levels were significantly lower. Minor disorders of the coagulation system were observed (Table 2). The imaging examinations performed at the Department of Pediatrics showed inflammatory changes in lung ultrasound (consolidations, pleural effusion), as well as significant leaks into the third space in the abdominal cavity.

The unusual similarity between both boys during the clinical course of the disease, as well as the changes in the laboratory and imaging tests, are noteworthy. For logistical reasons, the boys were placed in one patient room during hospitalization. They turned out to be friends from one kindergarten in the same group, where an outbreak of scarlet fever had been diagnosed a few days earlier.

Recognition of the disease as scarlet fever in case no. 2 was considered from the very beginning, while in case no. 1, due to the lack of characteristic symptoms within the first few days of hospitalization, the disease was only recognized after obtaining a throat swab and information from his medical history. After appropriate treatment was implemented, the condition of boy no. 1 improved rapidly (Table 2).

## 3. Discussion

In Poland, there is an obligation to report infectious diseases, infections, deaths, and biological pathogens to the state sanitary inspectorate. This includes scarlet fever. The epidemiological data prepared by the National Institute of Public Health (NIH)—National Research Institute indicate a significant increase in the number of incidents (per 100,000 inhabitants) of scarlet fever in 2022 (33.09) compared to the previous year (6.94). This is due to the reduced transmission of biological pathogens caused by the SARS-CoV-2 pandemic in 2021, which resulted in restrictions on the movement and attendance of children and young people in kindergartens and schools, as well as reduced access to stationary medical consultations during the pandemic. The highest number of cases in Poland was observed in the first quarter of the year. The statistical data show that 1415 scarlet fevers were diagnosed in Poland in the first half of 2021. This is an incidence rate of 3.71 per 100,000 inhabitants. In the first half of 2022, 5269 cases were already diagnosed, giving an incidence rate of 13.81 per 100,000 inhabitants. On the other hand, the highest number of cases by age falls in the age ranges of 0–4 and 5–9, with a higher incidence among boys. The number of hospitalizations resulting from scarlet fever in the discussed years did not exceed 2.5%, and no deaths caused by this disease were recorded [7,8].

Usually, the course of scarlet fever is mild, and a return to normal activity occurs after two days with the use of appropriate treatment; very rarely is it a reason for hospitalization [2].

In its classic course, scarlet fever remains a fairly simple disease to recognize. The patients described in this paper, however, were difficult cases to diagnose due to symptoms that could be indicative of several other childhood diseases. Patient no. 2 had the characteristic features: a distinctive rash, a Filatov’s triangle, a raspberry red tongue, and white coating. Patient no. 1 also had a confluent erythema on his face, and oral examination was limited due to severe inflammation of the gums, tongue, and lips, with significant damage to the mucous membranes.

Both patients gave the impression of “very sick children”. Their activity was significantly reduced, and the fever responded poorly to antipyretics. A big problem was the limitation of diuresis, causing peripheral edema of the feet, hands, and sacro-lumbar region. The fluid balance, which was maintained for several days, leveled off only after the use of diuretics. The patients had no record of family history of immunodeficiency or serious bacterial infections. The entire clinical picture gives grounds for considering the existence of a toxic form of *streptococcus pyogenes* infection.

Finally, the throat swab as the main standard enabled the final diagnosis of patient no. 1. The common source of infection, i.e., the kindergarten group and contact with a confirmed case of scarlet fever, left no doubt that it was indeed this disease. After the implementation of a dedicated treatment (phenoxymethyl penicillin), intensive parenteral hydration, diuretic treatment, and use of albumin specimens (in patient 1) with meticulous fluid balance, the boys’ clinical condition gradually improved. The patients were discharged in good general condition with recommendations after several days of hospitalization.

The possible impact of surviving a SARS-CoV-2 infection in children remains an open question. Patient no. 1 was infected with the Sars-Cov-2 virus shortly before the mentioned infection, and Patient no. 2 had a negative history. This information was confirmed by a screening test for the presence of antibodies to this virus. Taking into account the location of the Department of Pediatrics (Silesia region), the boys’ clinical condition, and the initial test results, PIMS-TS syndrome was suspected. Finally, the patients did not meet the criteria for diagnosis, and the treatment implemented was adequately effective.

Another disease that was initially considered was Epstein–Barr virus (EBV) infection with a severe course (a high temperature, cervical lymphadenopathy, hepatosplenomegaly, and swollen tonsils). Patient no. 1 tested positive in a heterophile antibody screening; however, both boys tested negative for EBV specific antibodies.

The etiology of acute pharyngitis is usually viral. In order not to unnecessarily administer an antibiotic, it is necessary to introduce differential diagnostics, such as pharyngeal cultures for bacteria. The leading agent in this group is *streptococcus pyogenes*. Diagnosis based solely on the clinical image can sometimes be quite difficult. This is because oropharyngeal lesions are not always present in positive cases of infection by GAS. Proper management avoids the development of the problem of antibiotic resistance in patients with GAS [5].

Both the CDC and the Canadian Pediatric Society have established criteria for *Group A Streptococcal* toxic shock syndrome. These patients meet the criteria for probable toxic shock [9,10]. In the literature, cases of toxic shock that are caused by *streptococcus pyogenes* can be found, in which mortality in the pediatric population can be very high, and complications are extremely dangerous [11,12].

Lamagni and colleagues investigated factors that may contribute to the generalization and severity of *S. pyogenes* infection. Based on cases in Greece, a conclusion was drawn about virological factors favoring a severe course of the disease, which may include chickenpox. A patient’s current condition should also be confronted with data about vaccinations and past infectious diseases [12,13,14].

## 4. Conclusions

Scarlet fever is generally a mild childhood disease, but the possibility of a severe course requiring hospitalization also exists. Therefore, this disease should not be marginalized by other infectious diseases. A diagnosis can be made on the basis of clinical symptoms, but these symptoms are not always clear. Differential diagnosis with other diseases is required. Medical history, which is often very detailed, can be the solution to making a definitive diagnosis and can complement the data collected from the laboratory tests performed.

## Figures and Tables

**Table 1:** Initial diagnosis in the emergency room.

Marked Laboratory Tests	Patient Number 1	Patient Number 2	Normal Ranges
Complete blood count			
WBC [×10^3^/µL (%)] Neut [×10^3^/µL (%)] Lymph [×10^3^/µL (%)] Mono [×10^3^/µL (%)] Eosy [×10^3^/µL (%)] Baos [×10^3^/µL (%)] RBC [×10^6^/µL] Hgb [g/dL] HCT [%] MCV [fL] MCH [pg] MCHC [g/dL] Rdw [%] PLT [×10^3^/µL] MPV [fl]	17.9 15.6 (87.2%) 0.4 (2.4%) 1.4 (7.9%) 0.2 (1.1%) 0.3 (1.5%) 4.47 12.5 35.2 78.7 27.9 35.5 11.3 322 5.4	10.6 8.4 (79.8%) 0.6 (5.4%) 1.2 (11.6%) 0.1 (1.0%) 0.2 (2.2%) 4.25 12.0 34.3 80.6 28.2 35.1 10.2 276 5.5	[4.0–10.0] [1.2–6.0] [1.0–5.5] [−1.0] [−0.7] [−0.1] [4.0–5.3] [11.5–14.5] [33.0–43.0] [76.0–90.0] [25.0–31.0] [32.0–36.0] [10.0–14.5] [150–400] [5.5–12.2]
CRP [mg/L]	290.68	165.7	[−5.0]
Procalcitonin [ng/mL]	51.6	5.0	[−0.5]
ALT [U/L]	37.5	196.3	[−41.0]
AST [U/L]	51.9	173.9	[−41.0]
Urea [mg/dL]	55.9	38.1	[16.6–48.5]
Creatinine [mg/dL] GFR [mL/min]	0.61 51	0.57 56.3	[0.7–1.2] [>60]
Sodium [mmol/L]	130	131	[135–145]
Potassium [mmol/L]	4.41	3.66	[3.5–5.1]
Capillary blood gases			
pH pCO2 [mmHg] O2 [mmHg] BE	7.40 26.2 61.6 −8.6	7.42 33.7 73.5 −2.9	[7.3–7.4] [35.0–45.0] [65.0–100.0] [−2–+2]
General examination of urine			
Color Leukocytes Ketone bodies RBCs	Cloudy urine 0–2 HPF ++ -	Cloudy urine 5–10 HPF - 2–4 HPF Single hyaline cast HPF Single bacteria HPF	Light yellow color <5 HPF - -

Abbreviations: WBC—white blood count total; Neut—neutrophils total; Limf—total lymphocytes; Mono—monocytes; Eosy—eosinophils; Baso—basophils; RBC—total red blood count; Hgb—hemoglobin concentration; Hct—hematocrit; MCV—mean corpuscular volume; MCH—mean corpuscular hemoglobin; MCHC—mean corpuscular hemoglobin concentration; Rdw—red blood cell distribution width; Plt—total platelet count; MPV—mean platelet volume; and RBCs—red blood cells.

**Table 2:** Diagnostics during hospitalization in the Department of Pediatrics.

Marked Laboratory Tests	I Control		II Control		III Control	
	Patient Number 1 (3rd Day of Hospitalization)	Patient Number 2 (2nd Day of Hospitalization)	Patient Number 1 (4th Day of Hospitalization)	Patient Number 2 (4th Day of Hospitalization)	Patient Number 1 (9th Day of Hospitalization)	Patient Number 2 (10th Day of Hospitalization)
Complete blood count						
WBC [×10^3^/µL (%)] Neut [×10^3^/µL (%)] Hgb [g/dL] PLT [×10^3^/µL]	17.7 9.1 11.1 181	9.7 6.0 (61.7%) 10.9 g/dL 226	14.1 3.8 (26.6%) 11.2 152	10.4 4.5 (43.4%) 10.61 243	18.2 11.8 (65.1%) 12.6 l 412	10.6 5.2 (49.0%) 12.4 514
CRP [mg/L]	178.7	103.86	87.45	71.4	43.9	39.0
PCT [ng/mL]	18.8	2.2	6.7	1.8	3.3	0.5
ALT [U/L]	33.9	116.3	32.7	90.5	33.8	77.2
AST [U/L]	40.4	53.6	32.9	48.3	36.6	44.4
Creatinine [mg/dL] GFR [mL/min]	<0.4 79.9	0.38 84.5	0.24 129.8	0.39 82.3	0.24 129.8	0.33 97.3
Urea [mg/dL]	30.1	22.4	20.1	23.1	18.6	23.0
Total protein [g/dL]	5.59	5.02	5.46	5.21	5.69	7.68
Total cholesterol [mg/dL]	99	140	101	204	172	180
Triglycerides [mg/dL]	307	492	338	442	342	223
LDL fraction [mg/dL]	9	13	10	22	118	150
HDL fraction [mg/dL]	14	14	13	12	23	36
Fibrinogen [g/L]	4.9	4.6	2.7	3.0	2.1	2.5
APTT [sec]	27	31	26	29	29	27
INR	0.9	1.2	1.0	1.1	1.0	1.0
D-dimer [mg//]	1.6	1.14	1.9	5.19	0.8	0.7
Troponin T [pg/mL]	4.9	4.7	4.8	4.8	4.7	4.4
CK-MB [U/L]	20.4	19.6	23.6	21.5	19.8	21.9
NT-proBNP	1178	995	-	-	425	-
Albumin [g/dL]	3.14	2.9	-	-	3.0	-

Abbreviations: WBC—total white blood count total; Neut—total neutrophils; Hgb—hemoglobin concentration; Plt—total platelet count; and MPV—mean platelet volume. I Control refers to laboratory tests performed between 24 and 48 h of hospitalization. II Control refers to tests performed again on the 4th day of hospitalization. III Control refers to tests performed at the final stage of hospitalization on day 9 or 10.

## Data Availability

All relevant data are within the manuscript.
